# Terpenoids in Marine Heterobranch Molluscs

**DOI:** 10.3390/md18030162

**Published:** 2020-03-14

**Authors:** Conxita Avila

**Affiliations:** Department of Evolutionary Biology, Ecology, and Environmental Sciences, and Biodiversity Research Institute (IrBIO), Faculty of Biology, University of Barcelona, Av. Diagonal 643, 08028 Barcelona, Spain; conxita.avila@ub.edu

**Keywords:** marine natural products, Gastropoda, chemical ecology, bioactivity

## Abstract

Heterobranch molluscs are rich in natural products. As other marine organisms, these gastropods are still quite unexplored, but they provide a stunning arsenal of compounds with interesting activities. Among their natural products, terpenoids are particularly abundant and diverse, including monoterpenoids, sesquiterpenoids, diterpenoids, sesterterpenoids, triterpenoids, tetraterpenoids, and steroids. This review evaluates the different kinds of terpenoids found in heterobranchs and reports on their bioactivity. It includes more than 330 metabolites isolated from ca. 70 species of heterobranchs. The monoterpenoids reported may be linear or monocyclic, while sesquiterpenoids may include linear, monocyclic, bicyclic, or tricyclic molecules. Diterpenoids in heterobranchs may include linear, monocyclic, bicyclic, tricyclic, or tetracyclic compounds. Sesterterpenoids, instead, are linear, bicyclic, or tetracyclic. Triterpenoids, tetraterpenoids, and steroids are not as abundant as the previously mentioned types. Within heterobranch molluscs, no terpenoids have been described in this period in tylodinoideans, cephalaspideans, or pteropods, and most terpenoids have been found in nudibranchs, anaspideans, and sacoglossans, with very few compounds in pleurobranchoideans and pulmonates. Monoterpenoids are present mostly in anaspidea, and less abundant in sacoglossa. Nudibranchs are especially rich in sesquiterpenes, which are also present in anaspidea, and in less numbers in sacoglossa and pulmonata. Diterpenoids are also very abundant in nudibranchs, present also in anaspidea, and scarce in pleurobranchoidea, sacoglossa, and pulmonata. Sesterterpenoids are only found in nudibranchia, while triterpenoids, carotenoids, and steroids are only reported for nudibranchia, pleurobranchoidea, and anaspidea. Many of these compounds are obtained from their diet, while others are biotransformed, or *de novo* biosynthesized by the molluscs. Overall, a huge variety of structures is found, indicating that chemodiversity correlates to the amazing biodiversity of this fascinating group of molluscs.

## 1. Background

Marine organisms produce a wide variety of natural products, often unique and critical for their survival and ecological performance [1,2,3,4]. Among these molecules, terpenes are remarkably abundant, with about 60% of all known natural products being terpenoids [1,2]. Terpenoids (or isoprenoids) are a large and diverse class of naturally occurring organic chemicals derived from terpenes; they are the largest class of natural products, with estimates of > 70 000 distinct compounds providing a vast pool of complexity that can interact with biological targets in a huge variety of ways [5]. They usually are multicyclic structures with oxygen-containing functional groups. Biochemical modifications of terpenes produce the related terpenoids. Terpenes are biosynthetically derived from units of isopentenyl pyrophosphate (IPP). Animals produce terpenes through the HMG-CoA reductase pathway, the mevalonate pathway, which also produces cholesterol. These reactions take place in the cytoplasm of the cells, where IPP and dimethylallyl pyrophosphate (DMAPP) condense to produce geranyl pyrophosphate (GPP), the precursor to monoterpenes and monoterpenoids (C10), and in fact to all terpenes and terpenoids. IPP is isomerized to DMAPP by the enzyme isopentenyl pyrophosphate isomerase. GPP is also converted to farnesyl pyrophosphate (FPP) and geranylgeranyl pyrophosphate (GGPP), respectively, C15 and C20 precursors to sesquiterpenes and diterpenes (as well as sesquiterpenoids and diterpenoids), and so on [6]. Biosynthesis is mediated by terpene synthases and further includes secondary transformations to produce modifications of the basic parent skeletons generating the astonishing variety of different terpenoids, usually involving oxidation, reduction, isomerization, and conjugation reactions, which provide specific functional properties to the terpenoids [7,8]. Thus, terpenoids are usually classified according to the number of C, based on the isoprene unit (C5), that comprise the parent terpene [9,10]. Terpenoids exhibit highly specialized biological activities ranging from nerve regeneration to blood-sugar regulation [11]. While terrestrial terpenoids have been known and used by humans for centuries, marine terpenoids are still understudied. Marine terpenoids are reported in annual reviews on the isolation of new C10, C15, C20, and C30 isoprenoids and sterols, and there is a wealth of literature dealing predominantly with marine monoterpenoids, diterpenoids, sesterterpenes, sterols, and others [12]. Some studies report the presence and potential significance of terpenoids occurring in marine invertebrates, while others deal with their synthesis [11,12]. 

The methodology for extracting natural compounds from organisms includes diverse techniques that are usually expensive and time-consuming [13,14]. Some studies have recently dealt with different methodologies and their advantages, and in particular, with new extraction techniques, including selective extraction of marine natural products, some of which are useful for terpenoids [13,14]. No studies, however, have used these new methods in heterobranchs, as far as we know [13].

**Figure 1:**
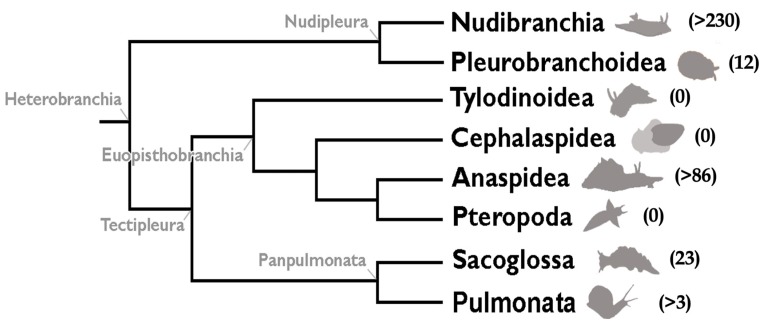
Schematic representation of the phylogeny of Heterobranchia, adapted and simplified from [3,19,20]. In brackets: number of terpenoids reviewed here.

Within marine organisms, molluscs are one of the most abundant and chemically rich invertebrate groups, possessing high amounts of terpenoids [3,15,16]. Heterobranch molluscs are widespread animals, living in all oceans, from the tropics to the poles, and from shallow waters to deep basins [3]. Most heterobranchs are soft-bodied and shell-less animals possessing a huge array of defensive strategies, from behavioral, morphological (mechanical or physical), as well as chemical strategies [15]. They comprise the commonly known sea slugs and sea hares, as well as marine pulmonates. In the past, Gastropod molluscs were divided into three subclasses: Prosobranchia, Opisthobranchia, and Pulmonata, because of the position and type of their respiratory structures. However, this is not considered valid anymore and currently Heterobranchia include the old “opisthobranch” gastropods as well as their marine pulmonate relatives (Figure 1) [17,18,19,20]. Heterobranchia comprises more than 8400 species [21], from which less than 300 have been chemically studied [1,2,3,15,22]. This is an evolutionarily old group of animals—from the Paleozoic era—therefore, a serendipitous morphological and ecological diversification can be observed, with many relevant adaptations in feeding modes, reproduction, and defensive strategies, among others [18]. Heterobranchs possess a wide variety of bioactive molecules, which protect them against potential predators and competitors, enhancing their ecological performance, being involved in reproduction, development, growth, or feeding, and affecting species distribution, community structure, and biodiversity [3,23]. Their chemical defenses may include natural products directly obtained from their prey, transformed from diet, or *de novo* biosynthesized. Among these chemicals, terpenoids are widely present.

Heterobranchs currently comprise eight major taxa, namely, Nudibranchia, Pleurobranchoidea, Tylodinoidea, Cephalaspidea, Anaspidea, Pteropoda, Sacoglossa, and Pulmonata [17,18,19,20] (Figure 1). Nudibranchs, or sea slugs, include four major taxa (Doridacea, Dendronotida, Euarminida, and Aeolidida), and comprise the most diverse group within Heterobranchia, with a huge variety of biological and chemical defensive strategies. Doridacea generally feed upon sponges, bryozoans, tunicates, or other “opisthobranchs”. They possess mainly terpenoids, either from their diet or *de novo* biosynthesized, but also alkaloids and other compounds [3,15]. Dendronotids predate either on cnidarians or certain small animals (crustaceans and turbellarians). Euarminida may prey upon octocorals or bryozoans. Aeolidida are mainly cnidarian-feeders usually protected by nematocysts from their prey (relocated into their dorsal cerata) but they also contain relevant chemicals [15]. Pleurobranchoidea include ascidian predators and scavengers. They often exude sulfuric acid at very high concentrations [24,25,26]. Tylodinoidea, or false limpets, feed upon sponges. Cephalaspideans, or head-shielded slugs and snails, present mainly polyketides and polyacetates, some of which are *de novo* biosynthesized, biomodified, or accumulated from their prey. Most of them are algal feeders, but some of them are active predators of other “opisthobranchs” (including other cephalaspideans), as well as annelid worms and sponges [15]. Anaspideans, or sea hares or aplysiomorphs, produce ink and other secretions and are herbivorous. They tend to accumulate all sorts of compounds from their diet. Pteropods include pelagic heterobranchs feeding on phytoplankton or preying upon other pteropods. Sacoglossans are usually cryptic and are very specialized herbivores, feeding upon different algal groups. They possess many sesquiterpenoids and diterpenoids (often acyclic), either from their diet or *de novo* biosynthesized, in addition to alkaloids and other compounds [3,15,27]. Marine pulmonates live at the intertidal and possess a wide array of propionates and terpenoids. 

Overall, heterobranchs display a huge diversity of biological and ecological strategies based on their chemical defenses. The presence of symbiotic microorganisms, which could in fact be producing some of these natural compounds, is unknown for this group [3]. The natural products of heterobranchs, however, are pivotal for their ecological specialization, as said above, although many aspects of their chemical ecology, as in other marine organisms, remain currently unknown [3,23,28,29]. As already mentioned, heterobranchs may feed upon a wide range of other organisms, such as algae (Chlorophyta, Ochrophyta, Rhodophyta (green, red, and brown algae, respectively)), sea grasses, Porifera, Cnidaria, Annelida, Bryozoa, Chordata (tunicates), other Mollusca, and others. Therefore, they are relevant in marine ecosystems because they occupy many different ecological niches and display a wide array of trophic relationships with organisms from many different taxonomic groups, comprising macroalgal or plant herbivory, carnivore prey-predator relationships, and occasionally cannibalism. They are also able to use cleptochemistry, incorporating chemicals from their diet (cleptochemicals), often called cleptochemodefenses when used for their own defensive means [15,30,31,32]. 

Marine organisms are a still underexplored source of unique natural compounds produced or accumulated both by micro- and macroorganisms with pharmacologically interesting properties to be used as drugs [1,2,23,33,34,35], displaying specific biological activities and unique skeletons, rarely found in non-marine organisms [36,37,38,39,40]. The pharmacological potential of terpenoids from marine organisms has been reviewed in the past [12]. For heterobranchs, their pharmacological potential in drug discovery is remarkable. Promising heterobranch compounds include several aplyronines and dolastatins from the anaspidean *Dolabella auricularia* and bursatellanins from *Bursatella leachii*, kahalalides from the sacoglossan *Elysia rufescens,* jorumycin from the doridacean *Jorunna funebris*, ulapualides from *Hexabranchus sanguineus*, and kabiramide from *Hexabranchus* sp, some of them being examples of highly effective compounds against tumors in clinical trials [39,41,42,43]. 

A detailed overview of the natural products involved in chemical defense in Heterobranchs was published recently, adding to previous reviews on heterobranch molluscs [3,15]. The review included a detailed discussion on the different kinds of compounds found in different groups, as well as on the origin and anatomical allocation of these chemical defenses, their biosynthesis, biogeography, and evolutionary patterns. These aspects will not be evaluated here. In addition, previous reviews covered the literature on “opisthobranch” chemistry and chemical ecology in particular geographical areas, in selected groups of compounds, or for certain heterobranch groups [27,44,45,46,47,48,49,50,51,52,53]. Other wide reviews have also been published regarding molluscs, general marine chemical ecology, or marine chemistry [23,28,33,50,54,55,56,57,58,59,60]. Blunt and collaborators [1,2] have been regularly providing accurate reports of new marine natural products described, with many terpenoids being found every year. Therefore, a specific review on terpenoids in heterobranch molluscs seemed timely and necessary. The aim of this review is to present an overview of the main types of terpenoids found in heterobranch molluscs and the groups in which are they found, even if their bioactivity has not yet been described. There are more than 330 molecules fully referenced in the original papers, and the intention here is to show examples of each type to illustrate their variety, for which the molecular structures have been reported. This review does not cover data regarding synthesis of the compounds. The described compounds are included here according to the type of terpene, even though some carbons may have been added or lost; compounds already reported in previous reviews will not be repeated here, except for relevant new information about them. Thus, this review mostly includes selected data published over the last 25 years [3,15]. It is remarkable that no terpenoids have been described in this period in tylodinoideans, cephalaspideans, or pteropods (Figure 1). Most terpenoids have been found in nudibranchs, anaspideans and sacoglossans, and very few in pleurobranchoideans and pulmonates, as reported below. 

## 2. Monoterpenoids

Monoterpenes are formed by two isoprene units and have a molecular formula C_10_H_16_. They may be linear (acyclic) or contain rings, and are derived biosynthetically from units of IPP, which is formed from acetyl-CoA via mevalonic acid in the HMG-CoA reductase pathway. IPP is further isomerized to DMAPP by the enzyme IPP isomerase. GPP is the precursor to monoterpenes, and their biosynthesis is mediated by terpene synthases [6,7,8]. Within heterobranchs, in this time period, only anaspideans and sacoglossans are found to possess monoterpenoids, which may be linear or cyclic (Table 1). When cyclic monoterpenoids are present (in sea hares and one sacoglossan), these are always monocyclic.

### 2.1. Linear Monoterpenoids

**Figure 2:**
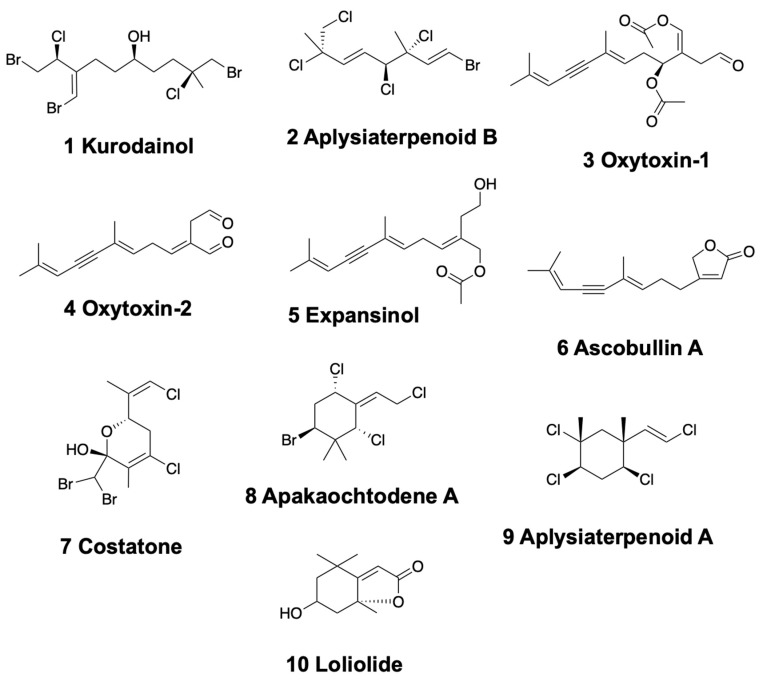
Structures of selected monoterpenoids from Heterobranch molluscs: (**1**) kurodainol; (**2**) aplysiaterpenoid B; (**3**) oxytoxin-1; (**4**) oxitoxin-2; (**5**) expansinol; (**6**) ascobullin A; (**7**) costatone; (**8**) apakaochtodene A; (**9**) aplysiaterpenoid A; (**10**) loliolide.

Anaspideans and sacoglossans possess several kinds of linear monoterpenoids, either from their diet or transformed from dietary compounds. Within anaspideans, the sea hare *Aplysia kurodai* from Japan presents halogenated and brominated monoterpenoids distributed in different body parts, such as kurodainol (1) [61] and aplysiaterpenoid B (2) (Figure 2) [62,63]. Mediterranean specimens of *Aplysia fasciata* contain polyhalogenated monoterpenes similar to those of *Plocamium* red algae [64]. These compounds include the 3,4-erythro-7-dichloromethyl-3-methyl-3,4,8-trichloro-1,5 (E), 7 (Z)-octatriene, previously found in the Pacific *Plocamium cartilagineum*, and 3,4-threo-7-dibromomethyl-3-methyl-3,4,8-trichloro-1,5 (E), 7 (E)-octatriene [64]. Some of these chemicals were also present in the red algae *Plocamium coccineum*. Moreover, the mucus secretions of the Atlantic and Mediterranean Sea hare *Aplysia punctata* contain several *P. coccineum* halogenated monoterpenes too [65,66]. More specifically, these are four unusual acetates of linear polyhalogenated monoterpenes and four cyclic derivatives.

Some shelled sacoglossans are also rich in monoterpenoids, which they can obtain from their algal food and further transform into more bioactive compounds. The Mediterranean species *Oxynoe olivacea*, *Lobiger serradifalci,* and *Ascobulla* (*= Cylindrobulla*) *fragilis* feed on different parts of the green algae *Caulerpa prolifera*, which contains the sesquiterpenoid caulerpenyne [67]. *O. olivacea* and *A. fragilis* are able to modify caulerpenyne, which can be found in their digestive gland, into the potent ichthyotoxic aldehydes, oxytoxin-1 (3) and -2 (4) (Figure 2), and transport them to be secreted to the mucus and mantle. *L. serradifalci* instead contains only oxytoxin-1 in the parapodial lobes and in its defensive mucus [68]. Similarly, in the Caribbean species *Ascobulla ulla* (feeding on *Caulerpa fastigiata*), *Oxynoe antillarum* (feeding on *Caulerpa* sp.), and *Lobiger souberveii* (feeding on *Caulerpa racemosa*), the same chemistry is found, accumulating caulerpenyne (only this molecule is found in *L. souberveii*) and further transforming it into oxytoxins [69]. In the Indian species *Volvatella sp*. caulerpenyne has also been reported [70]. Caulerpenyne is a highly bioactive molecule, presenting anticancer activity, cell grown inhibition, neurotoxic activity, and inhibiting lipoxygenases, among others [71,72,73,74].

The shell-less sacoglossans of the genus *Elysia* usually also feed on *Caulerpa* species and related green algae [75], from which they obtain and transform their defensive compounds. Some Caribbean species of *Elysia*, such as *E. subornata* (feeding on *Caulerpa prolifera*), *E. patina*, and *E. nisbeti* (feeding on *Caulerpa* sp.), contain caulerpenyne and oxytoxin-1 [69]. *Elysia* cf. *expansa* from India also presents caulerpenyne, together with minor amounts of certain reduced derivatives, dihydrocaulerpenyne and expansinol (5) (Figure 2) [76], similar to the Mexican *Ascobulla ulla* reported above.

### 2.2. Monocyclic Monoterpenoids

Several anaspideans present monocyclic monoterpenoids obtained from red or brown algae [3]. *Aplysia parvula* accumulates several brominated and chlorinated compounds from the red alga *Plocamium costatum* in New Zealand. Among them, costatone (7) (Figure 2) is found to be 14 times more concentrated in the sea hare than in the red alga [77,78]. Fimbrolide is also found in *A. parvula* from Tasmania, where it feeds upon the red algae *Laurencia filiformis* [79]. On Guam, instead, *A. parvula* feeds on the red alga *Portieria hornemanii*, sequestering halogenated monoterpenes, apakaochtodene A (8) and B (Figure 2) and using them to deter potential fish predators [80]. In Japan, *Aplysia kurodai* also possesses aplysiaterpenoid A (9) (Figure 2), as well as aplysiapyranoids A–D, with moderate cytotoxicities against Vero, MDCK, and B16 cells (IC_50_ = 19–96 μg/mL) [62,63]. *Dolabella auricularia*, which feeds on brown Dictyotaceae algae, presents (-)−loliolide (10) (Figure 2) in the Indian Ocean but not in the Gulf of California [81,82,83,84]. Loliolide is considered to be a degraded carotenoid from dietary algae. 

In sacoglossa *Ascobulla ulla*, in contrast with the previously mentioned *Ascobulla* species, ascobullin A (6) and B (Figure 2) replace oxytoxins, being structurally related less reactive molecules. 

## 3. Sesquiterpenoids

Sesquiterpenoids are formed by three isoprene units and have molecular formula C_15_H_24_. They may be acyclic or contain rings, displaying many unique combinations. The reaction of GPP with IPP results in 15-C FPP, an intermediate in their biosynthesis [6]. Cyclic sesquiterpenes are more common than cyclic monoterpenes because of the increased chain length and the additional double bond in sesquiterpene precursors. The FPP backbone can be rearranged in several different ways and further complemented with different functional groups, thus producing a wide variety of sesquiterpenoids. Within heterobranchs, in this time frame, nudibranchs, anaspideans, sacoglossans, and pulmonates, have been found to possess sesquiterpenes, which are mostly cyclic, while no sesquiterpenoids have been found in Pleurobranchoidea or other heterobranchs (Table 2).

### 3.1. Linear Sesquiterpenoids

The doridacean *Actinocyclus papillatus* from South China presents actisonitrile in the mantle, a mildly cytotoxic compound [85,86], as well as actinofide, a terpenoid diacylguanidine [87].

### 3.2. Monocyclic Sesquiterpenoids

**Figure 3:**
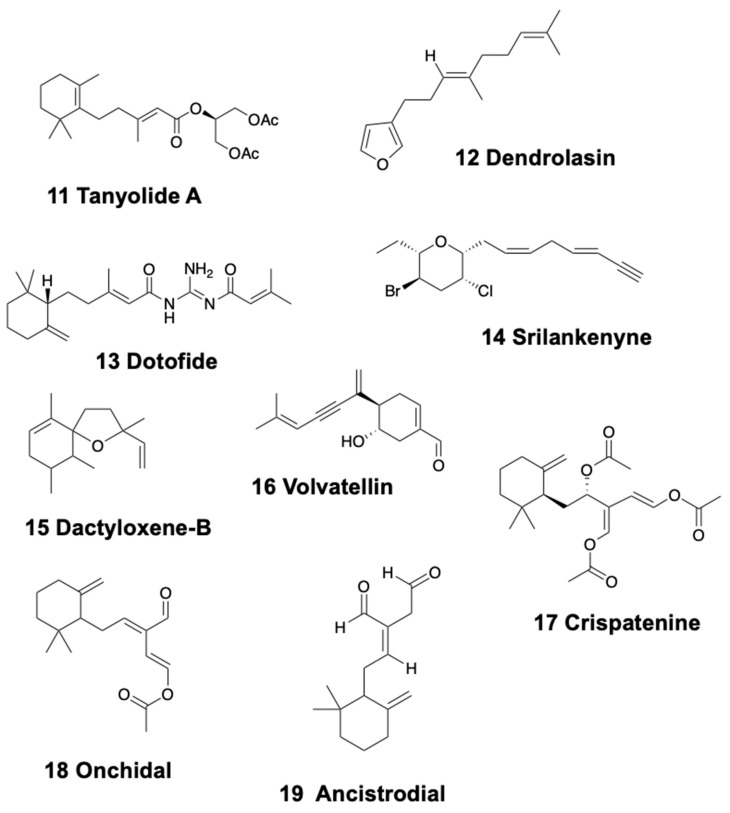
Structures of selected sesquiterpenoids from Heterobranch molluscs: (**11**) tanyolide A; (**12**) dendrolasin; (**13**) dotofide; (**14**) srilankenyne; (**15**) dactyloxene-B; (**16**) volvatellin; (**17**) crispatenine; (**18**) onchidal; (**19**) ancistrodial.

Nudibranchs, anaspideans, sacoglossa, and pulmonates present monocyclic sesquiterpenoids. In sea slugs, tanyolides A (11) and B (Figure 3) are found in the dorsal mantle of the doridacean *Sclerodoris tanya* from California, being used as effective fish-feeding deterrents [88]. In addition, some known sesquiterpenes, including dendrolasin (12) (Figure 3), are found in the Patagonian doridacean *Tyrinna nobilis* [89]. Furthermore, (5R,6Z)-dendrolasin-5-acetate was isolated from *Hypselodoris jacksoni* from Australia [90]. The dendronotid slug *Doto pinnatifida* from the Atlantic presents dotofide (13) (Figure 3), a guanidine-interrupted terpenoid with an unknown ecological role [91].

In sea hares, the Sri Lankan species *Aplysia oculifera,* possesses compounds obtained from the red algae *Laurencia*, such as srilankenyne (14) (Figure 3), while the digestive gland of Indic and West Pacific specimens presents brominated acetylenes instead [92,93]. *Aplysia argus* from Bahamas presents, among other compounds, the sesquiterpene ethers dactyloxene-B (15) (Figure 3) and dihydroxydeodactol monoacetate in whole body extracts [94,95,96,97,98,99].

The modified sesquiterpenoid, volvatellin (16) (Figure 3), was found in the Indian shelled sacoglossa *Volvatella sp*., along with the previously mentioned monoterpenoid, caulerpenyne (see above) [70]. The configurational assignment of volvatellin and its putative origin from oxytoxin-1 was further reported [100]. *Elysia crispata* from Venezuela, instead, was found to contain, among other compounds, crispatenine (17) and onchidal (18) (Figure 3), the latter also found in the pulmonate *Onchidella* [69,101,102]. The case of *E. crispata* is extremely intriguing because it includes different types of chemistry and chemical strategies: bioaccumulation, biotransformation, and biosynthesis. The pulmonate *Onchidella binneyi* contains onchidal, which is further secreted in its active form, ancistrodial (19) (Figure 3), to elicit deterrence against predators [103].

### 3.3. Bicyclic Sesquiterpenoids

**Figure 4:**
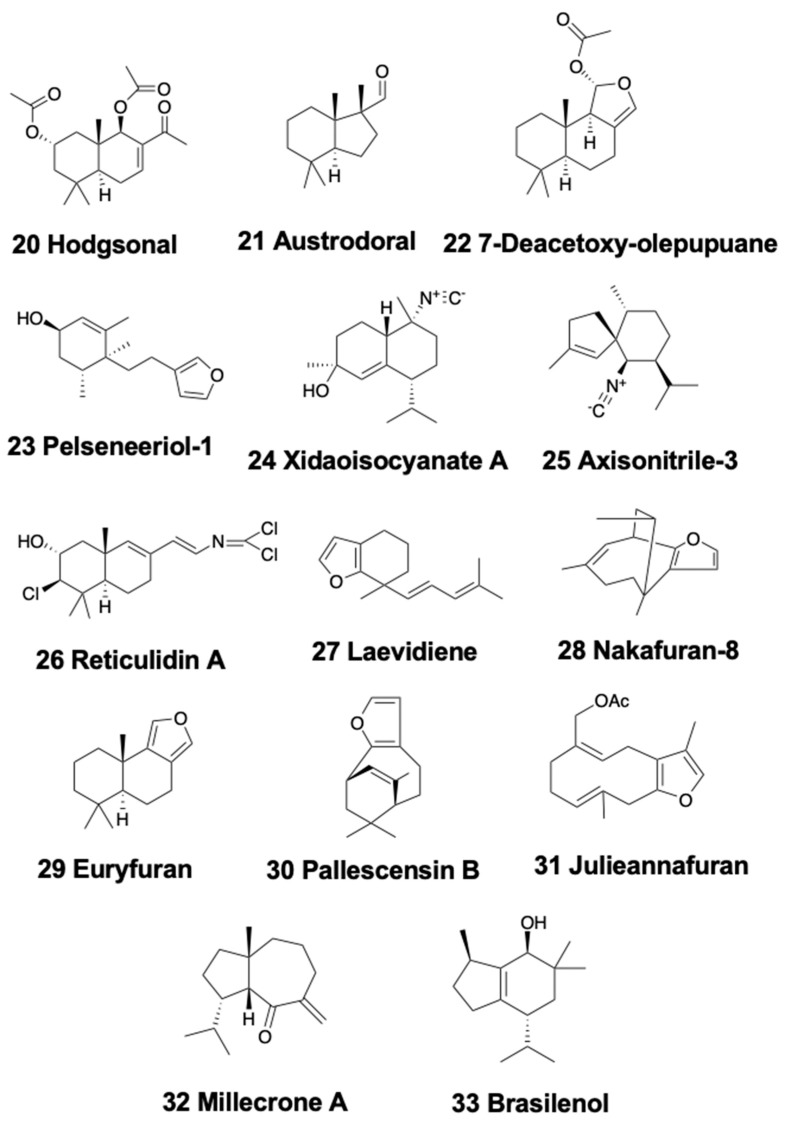
Structures of selected sesquiterpenoids from Heterobranch molluscs: (**20**) hogsonal; (**21**) austrodoral; (**22**) 7-deacetoxy-olepupuane; (**23**) pelseneeriol-1; (**24**) xidaoisocyanate A; (**25**) axisonitrile-3; (**26**) reticulidin A; (**27**) laevidiene; (**28**) nakafuran 8; (**29**) euryfuran; (**30**) pallescensin B; (**31**) julieannafuran; (**32**) millecrone A; (**33**) brasilenol.

Bicyclic sesquiterpenoids are mainly present in sea slugs, with a couple of examples in sea hares, and none in the remaining heterobranch groups. Within nudibranchs, the Antarctic doridacean *Bathydoris hodgsoni* contains the drimane sesquiterpene hodgsonal (20) (Figure 4) [104,105]. Hodgsonal is found in the most exposed body parts (mantle and dorsal papillae) and is probably *de novo* biosynthesized by the nudibranch. Hodgsonal is used as a feeding deterrent against sympatric predators, such as the sea star *Odontaster validus* and the anemone *Epiactis* sp. [105]. Furthermore, austrodorins A and B, and the two nor-sesquiterpenes austrodoral (21) (Figure 4) and austrodoric acid, were found in the Antarctic slug *Doris kerguelenensis*, but their ecological role is still unknown [106,107,108,109,110]. 

The widely studied nudibranch *Dendrodoris* possesses drimane sesquiterpenes distributed in different body parts [3,15]. In this slugs, drimane esters are generally associated to the reproductive organs and eggs, while drimane sesquiterpenes are detected in the mantle. *D. arborescens* possess the known sesquiterpene 7-deacetoxy-olepupuane (22) (Figure 4) [111], while *D. carbunculosa* presents several cytotoxic drimane sesquiterpenes, the dendrocarbins A–N [112]. Moreover, *D. krebsi* from Mexico also possess drimane sesquiterpenes and esters [113], allocated in the body in a way similar to that previously described for other *Dendrodoris* species [114]. *D. denisoni* from New Zealand presents cinnamolide, olepupuane, and polygodial in its mantle [78]. The phylogenetically related genus *Doriopsilla* also presents compounds related to those of *Dendrodoris*. Pelseneeriols-1 (23) and -2, two furanosesquiterpene alcohols, are present in the mantle of *Doriopsilla pelseneeri* from the Atlantic [115]. *De novo* biosynthesis of drimane esters, sesquiterpenes, and 15-acetoxy-ent-pallescensin was demonstrated for *D. areolata* and *Doriopsilla* sp. through the mevalonic pathway [116,117,118,119]. Two more diastereomeric acetates of pelseneeriol-1 and -2 (Figure 4) were further described in that study. Moreover, both *D. albopunctata* from the Pacific and *D. areolata* from the Atlantic also contain drimane sesquiterpenes and *ent*-pallescensin A [113], located in their bodies in a way similar to the *Dendrodoris* species [114]. *Doriopsilla pharpa* contains polygodial, and their extracts are deterrent to two fish, the blenny *Chasmodes bosquianus,* and the mummichog *Fundulus heteroclitus*, which learned to avoid food items that contained extracts from *D. pharpa* [120], while extracts of the slug were also rejected by the crabs *Callinectes similus* and *Panopeus herbstii* in the field. 

Another well-studied nudibranch group is that of the Phyllidids, which are often colorful animals containing isocyanate compounds [15]. These isocyanates display a wide array of activities, such as antifouling, antibiotic, antifungal, and antitumor properties, and have been studied in depth during recent years [121,122,123,124,125]. Several studies on dietary sesquiterpene isocyanides suggest that the nudibranchs sequester them from different demosponges, showing a much broader feeding variability than previously reported [46]. One of the most studied species is *Phyllidiella pustulosa,* where compounds are obtained from the demosponge *Acanthella cavernosa* [126]. Specimens from China and Vietnam also contain sesquiterpene isocyanides and related compounds, some of them found also in *Acanthella* sponges [126,127,128,129]. A recent chemical analyses of the South China Sea nudibranchs *Phyllidiella pustulosa* and *Phyllidia coelestis*, as well as their possible sponge prey *Acanthella cavernosa*, led to the isolation of a nitrogenous cadinane-type sesquiterpenoid, xidaoisocyanate A (24) (Figure 4), among other sesquiterpenoids and diterpenoids [130]. Moreover, *P. pustulosa* from Fiji presents an isothiocyanate, axisonitrile-3 (25) (Figure 4), and several minor related sesquiterpenes [131]. The isothiocyanate displays a moderated antiplasmodial activity, being weakly cytotoxic (IC_50_ > 20 μg/ml) but strong growth inhibitor of *Mycobacterium tuberculosis* (MIC 2 μg/ml) [132]. Furthermore, a sesquiterpene isonitrile was also isolated as an antifouling agent from the Japanese *P. pustulosa* [123], and some other studies on the antifouling potential of *Phyllidia ocelata*, *P. varicosa*, *Phyllidiella pustulosa,* and *Phillidiopsis krempfi*, found three more sesquiterpene isonitriles, the 10-epi-axisonitrile-3, 10-isocyano-4-cadinene, and 2-isocyanotrachyopsane, as well as a peroxide, 1,7-epidioxy-5-cadinene, and some more sesquiterpene isonitriles [122,133]. *P. pustulosa* and *Phyllidia ocellata* from Australia, also present some stereoisomers of 4-isocyano-9-amorphene and of 10-isocyano-4-amorphene, respectively, while *Phyllidia picta* from Bali contained the axane sesquiterpenoids pictaisonitrile-1 and pictaisonitrile-2 [134]. *Phyllidia* sp. from Sri Lanka also contains 3-isocyano-theonellin, closely related to a cyanide from the demosponge *Axinyssa* [135]. Some nitrogenous bisabolene sesquiterpenes from these species also possess a potent antifouling activity against barnacle larvae in *in vitro* assays [136,137]. *P. varicosa* presents two 9-thiocyanatopupukeanane sesquiterpenes, isolated as an epimeric mixture, found also in its demosponge prey *Axinyssa aculeata* [138]. One of these compounds is present in the mantle, suggesting an implication in chemical defense, while both are present in the digestive gland, supporting a dietary origin. Both compounds display mild toxicity against brine shrimp and some antimicrobial activity against *Candida albicans* and *Bacillus subtilis*. On the other hand, *P. coelestis* from Thailand also presents two cytotoxic pupukeanane sesquiterpenoids [139]. Several studies suggest all these compounds may play an important role in the chemical defense of phyllidids against fish predators, and that this is also related to visual defenses [3,140]. Contrastingly, *Reticulidia fungia* from Okinawa is different from the rest of the family members in presenting two sesquiterpenes of a rare class of sponge compounds, the cytotoxic carbonimidic dichlorides, reticulidins A (26) and B (Figure 4) [141]. Furthermore, the dorid *Hexabranchus sanguineus* from South China presents a couple of sesquiterpenes, as well as other compounds, suggested to originate from its sponge diet [142].

Regarding Chromodoridids, bicyclic sesquiterpenoids are also present in several species. For example, the Atlantic species *Cadlina laevis* and *C. pellucida* present laevidiene (27) (Figure 4), albicanol, and derivatives, some of them from the demosponge *Spongia agaricina,* on which they feed upon [15,143,144,145,146]. Furthermore, the spawn of *C. luteomarginata* from British Columbia also presents a drimane sesquiterpenoid [147], and the slug is able to biosynthesize some of its terpenoids [148]. The genus *Hypselodoris* (now *Felimida* for Eastern Pacific, Atlantic, and Mediterranean species) [149] is a good example of sponge-derived furanosesquiterpenes, usually accumulated into particular structures, called Mantle Dermal Formations (MDFs) [3,25]. *Hypselodoris picta webbi* from the NW Atlantic presents the furanosesquiterpenes longifolin and tavacfuran, while *Hypselodoris picta azorica* additionally presents microcionin-1 [150]. The South African *Hypselodoris capensis* presents several known sesquiterpenes, the antifeedant nakafurans 8 (28) and 9 (Figure 4), and several sesterterpenes (see below), from the sponges *Fasciospongia* sp. and *Dysidea* sp. on which it probably feeds [151]. Moreover, *H. infucata* from Hawai’i presents nakafuran 8 and 9, probably from *Dysidea fragilis* [152]. *H. obscura* from Australia presents dendrolasin, (−)-euryfuran, and (+)-pallescensin A, while *H. whitei* possesses (−)-euryfuran (29) (Figure 4), (−)-furodysin, (−)-furosydinin, and dendrolasin [90]. Some known sesquiterpenes, including dehydropallescensin-2 are found in the Patagonian doridacean *Tyrinna nobilis,* together with an unusual diterpene (see below) [89]. In addition, several species of the genus *Ceratosoma* from China contain bicyclic sesquiterpenoids. In particular, *Ceratosoma trilobatum* and *C. gracillimum* contain the four furanosesquiterpenes, pallescensin B (30) (Figure 4), (−)-furodysinin, (−)-dehydroherbadysidolide, and (−)-herbadysidolide, previously found in *Dysidea* spp. sponges, and suggested to be obtained from diet. These sea slugs possess an interesting mechanism with dorsal horns containing MDFs fully loaded with furanosesquiterpenoids. Although a defensive role is highly probable, it remains to be further proved using sympatric predators [46,153]. Recently, a study on 19 species of Chromodorididae nudibranchs from Australia reported 72 compounds, including many bicyclic furanosesquiterpenoids, among others, found in mantle and internal glands [154]. In particular, *Mexichromis festiva* contains euryfuran and dendrolasin, while *M. mariei* only possesses euryfuran. *Hypselodoris bennetti* and *H. obscura* both present euryfuran as major metabolite, but *H. bennetti* contains also agassizin, dehydroherbadysidolide, and pallescensone. *H. obscura* also presents furodysinin, furodysin, and dendrolasin, while *H. tryoni* contains dehydroherbadysidolide, furodysinin, nakafuran-9, and dendrolasin. In the same study, *Ceratosoma trilobatum* was found to present furodysinin, furodysin, and dendrolasin in the viscera, while other compounds were found additionally in the mantle: agassizin and dehydroherbadysidolide, and *C. brevicaudatum* possesses mixtures of the same metabolites and some unidentified compounds.

In Dendronotids, the species *Tritonia hamnerorum* from Florida sequesters julieannafuran (31) (Figure 4), a furanogermacrane from its food, the sea fan *Gorgonia ventalina*, which is deterrent against reef fish in field assays [155]. Furthermore, some byciclic sesquiterpenes are found in the mantle of *Tochuina tetraquetra* and its food, the soft coral *Gersemia rubiformis* from British Columbia [156]. These are tochuinyl acetate and dihydrotochuinyl acetate, two cuparane sesquiterpenoids, found together with some diterpenes (see below). Their ecological activity has not been studied so far. 

Similarly, the Arminacean *Leminda millecra* from South Africa presents several sesquiterpenes, probably from its octocoral prey (*Alcyonium foliatum*, *A. valdiviae*, *A. fauri*, and *Capnella thyrsoidea*) [157,158]. These are millecrones A (32) and B and millecrols A and B (Figure 4). Among them, millecrone A inhibited the growth of *Candida albicans* at 50 µg/disk, while millecrone B was active against *Staphylococcus aureus* and *Bacillus subtilis* at 50 µg/disk; millecrol B was active only against *B. subtilis* at 50 µg/disk [45]. In other sampling areas, *L. millecra* presents some quinones, and some of its chemicals are described to be obtained from its diet, the gorgonian *Leptogorgia palma* [158]. Again, their ecological activity has not been studied yet. In Japan, the arminid *Dermatobranchus otome* possesses germacrane sesquiterpenoids DO1, DO2, and DO3, with some antibacterial activity against *B. subtilis* [159]. 

Among Aeolids, *Phyllodesmium lizardensis* from Australia presents muurolene sesquiterpenes from its prey, the coral *Bayerxenia* (*Heteroxenia*) sp. [160]. In China, *P. magnum* presents a rare unnamed asteriscane sesquiterpene (C_15_H_24_), together with other sesquiterpenes [161], which is related to 11β-acetoxypukalide, the previously described defensive compounds of *P. guamensis* [162], thus suggesting a dietary origin from *Sinularia* soft corals.

Finally, in sea hare, *Aplysia argus* from South China presents a series of ethers, like those found in the red algae *Laurencia pinnatifida* and *L. obtusa*, together with some other enantiomers [163]. Moreover, the sesquiterpene ether (+)-brasilenol (33) (Figure 4), previously found in *L. obtusa* and *A. brasiliana*, is also present in *A. argus* [163]. The Atlantic *A. californica* transforms laurinterol and pacifenol from algal species of *Laurencia* and *Plocamium* through acid catalysis in its digestive gland, into the halogenated terpenoids aplysin and pacifidiene [164,165,166,167]. Three more sesquiterpenes, 6-hydroxy-1-brasilene, epibrasilenol acetate, and 6-*epi*-*β*-snyderol, as well as one acetogenin, (3Z, 9Z)-7-chloro-6-hydroxy-12-oxo-pentadeca-3,9-dien-1-yne, and other compounds, are found in *Aplysia fasciata* from the Mediterranean [168].

### 3.4. Tricyclic Sesquiterpenoids

**Figure 5:**
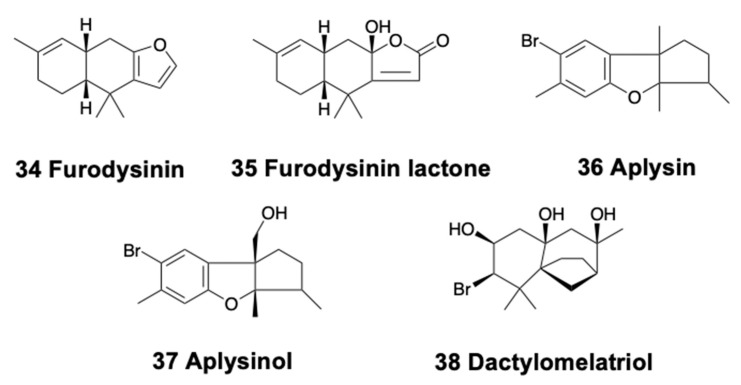
Structures of selected tricyclic sesquiterpenoids from Heterobranch molluscs: (**34**) furodysinin; (**35**) furodysinin lactone; (**36**) aplysin; (**37**) aplysinol; (**38**) dactylomelatriol.

Only nudibranchs and sea hares present tricyclic sesquiterpenoids. Pallescensin A and several other terpenes are found in the Patagonian doridacean *Tyrinna nobilis* [89]. *Hypselodoris infucata* from Bali yielded the known (−)-furodysinin (34) (Figure 5), being active against the HeLa cell line with an IC_50_ at 102.7 μg/mL, while its crude extract is repellent to the sympatric shrimp *Penaeus vannamei* at natural concentration [169]. Similarly, the species *H. kanga* from India possesses furodysinin, suggesting a trophic relationship, since furodysinin is also found in the associated demosponge *Dysidea* sp. [170]. *H. lajensis* from Brazil contains furodysinin lactone (35) (Figure 5), also found in *Dysidea* sponge species [171]. *Goniobranchus reticulatus* from Australia presents a dialdehyde sesquiterpene, together with the ring-closed acetal, both bioactive against P388 mouse leukemia cells, as well as some diterpenes [90]. These two compounds are also found in *Goniobranchus sinensis* (previously *Chromodoris sinensis*) from China, where they are reported to be deterrent [90].

Two brominated compounds, aplysin (36) and aplysinol (37) (Figure 5), are found in *Aplysia kurodai* from Japan [172]. This species is also a source of several alkaloids and other compounds [3]. On the other hand, dactylomelatriol (38) (Figure 5) is found in *Aplysia dactylomela* from the Atlantic, derived from an omphalane skeleton and previously described only in terrestrial fungi [173]. Dactylomelatriol is suggested to originate by a modification of a precursor obtained from *Laurencia* red algae. In fact, the sea hare *A. dactylomela* is one of the most prolific sources of natural products, containing mixtures of compounds, including many sesquiterpenes, along with polyketides, diterpenes, and triterpenes, which are usually biotransformed from red algal compounds. This species is now considered to be in fact two species: *A. dactylomela* and *A. argus*, from the Atlantic and Indo-Pacific, respectively [174]. The dietary compounds found in these sea hares display very diverse structures and characteristics and have been recently reviewed [3].

## 4. Diterpenoids

Diterpenoids are composed of four isoprene units (two terpene units) and usually have the molecular formula C_20_H_32_. They are biosynthesized via the HMG-CoA reductase pathway, with geranylgeranyl pyrophosphate (GGPP) being a primary intermediate; structural diversity is achieved mainly by diterpene synthases [6]. Diterpenoids in heterobranchs often appear within mixtures of compounds, including other terpenes or even structurally different compounds, and are mostly found in nudibranchs (Table 3). In addition, many halogenated and brominated monoterpenes and diterpenes are allocated into different body parts in sea hares, for example in *Aplysia kurodai* (see below). 

### 4.1. Linear Diterpenoids

**Figure 6:**
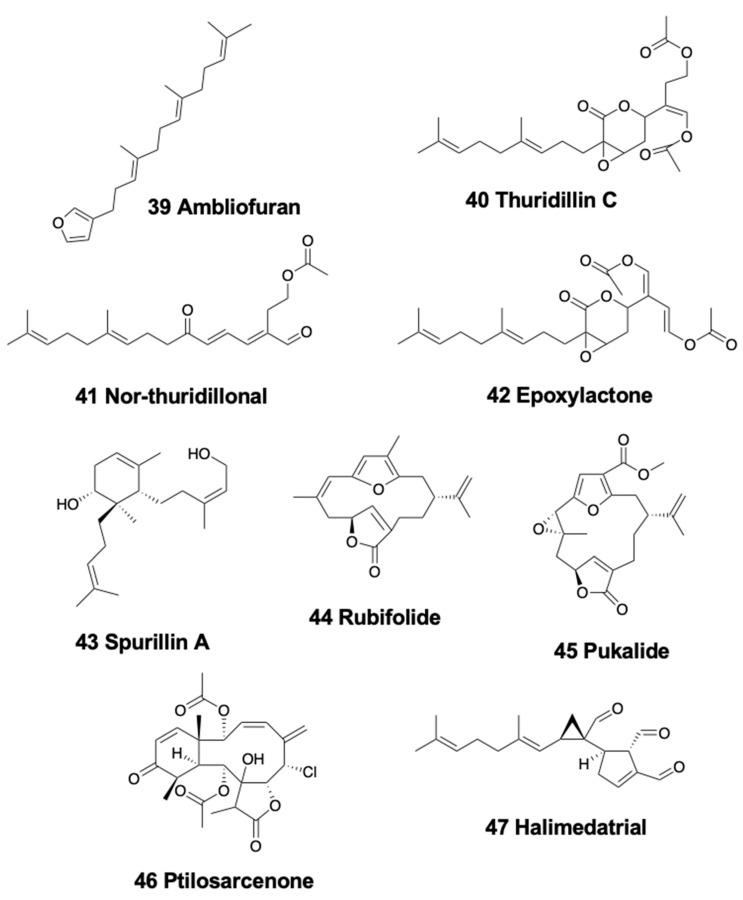
Structures of selected diterpenoids from Heterobranch molluscs: (**39**) ambliofuran; (**40**) thuridillin C; (**41**) nor-thuridillonal; (**42**) epoxylactone; (**43**) spurillin A; (**44**) rubifolide; (**45**) pukalide; (**46**) ptilosarcenone; (**47**) halimedatrial.

Among the sea slugs, the chromodorid nudibranch *Goniobranchus splendidus* from Australia presents 31 different terpenes, including the linear furanoditerpene ambliofuran (39) (Figure 6) [154], while *Chromodoris reticulata* (also from Australia) presents this compound in its viscera [175].

In sacoglossans, only shell-less species contain linear diterpenoids. The Mediterranean *Thuridilla hopei*, a beautifully colored species, contains the three diterpenoids thuridillins A–C (40) (Figure 6) [176] and three nor-thuridillonals—nor-thuridillonal (41), dihydronor-thuridillonal, deacetyl-dihydro-nor-thuridillonal (Figure 6)—suggested to derive from the common dietary precursor epoxylactone (42) (Figure 6) [177]. *T. hopei* feeds and lives on the caulerpal green alga *Derbesia tenuissima* [178], also containing the active diterpenoid epoxylactone. The related compounds thuridillins D-F are also present in the Australian species *T. splendens* [179]. Thuridillins from *T. splendens* did not deter feeding by the sympatric shrimp *Palaemon serenus* [90]. The conversion of the precursors to thuridillins and nor-thuridillonals is suggested to be a detoxification mechanism by oxidation or reduction processes [176,177]. Contrastingly, *Elysia translucens* presents udoteal from the green algae *Udotea* [178,180,181].

### 4.2. Monocyclic Diterpenoids

Only nudibranchs, sea hares, and sacoglossans are found to possess monocyclic diterpenoids in the period reviewed here. Included in the mixture of terpenes found in *Phyllidiella pustulosa* from China is one monocyclic isocyanide diterpenoid [127]. The species *Phyllodesmium longicirrum* from Australia presents cembranoid diterpenes obtained from its prey, the soft coral *Sarcophytum trochelioforum* [182]. The chemical study of *Spurilla* aeolids led to the finding of terpenoid compounds, spurillin A (43) (Figure 6) from the mediterranean *S. neapolitana*, and (−)-cis-*γ*-monocyclofarnesol from the argentinian *Spurilla* sp., together with other compounds [183]. *Tochuina tetraquetra* from British Columbia presents two previously known cembrane diterpenoids, rubifolide (44) and pukalide (45) (Figure 6), together with some sesquiterpenes, obtained from the soft coral *Gersemia rubiformis*, with unknown role [156]. *T. tetraquetra* also presents briarein-type diterpenoids, such as ptilosarcenone (46) (Figure 6) and deacetyl ptilosarcenone butanoate [156].

The sea hare *Aplysia depilans* presents nine brominated diterpenes with the rare dactylomelane skeleton, previously described in *A. dactylomela* [184]. The 16-acetoxy-15-bromo-7-hydroxy-9(11)-parguerene is found in *Aplysia fasciata* from the Mediterranean [168].

Within sacoglossans, the Mediterranean species *Bosellia mimetica* possesses the diterpenoid halimedatrial (47) (Figure 6) from its prey, the green algae *Halimeda tuna* [178]. Halimedatetraacetate is its inactive form, being quite similar to caulerpenyne (from *Caulerpa* algae), except for possessing 20 instead of 15 C atoms [180,185,186,187]. Other sacoglossans also present similar strategies, for example the Caribbean species *Elysia pusilla* (formerly *E. halimedae*) incorporates halimedatetraacetate and halimedatrial from *Halimeda macroloba* [186], and *E. tuca* obtains halimedatetraacetate from *Halimeda incrassata* [69], while the species *E. translucens* presents the related metabolite udoteal, as mentioned above [178,180,181].

### 4.3. Bicyclic Diterpenoids

**Figure 7:**
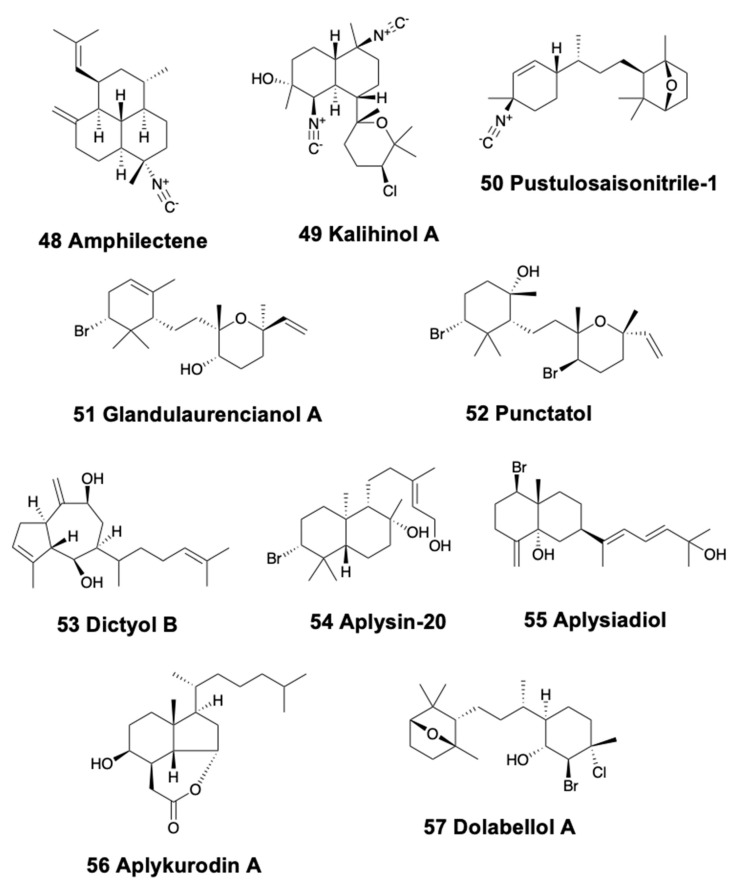
Structures of selected diterpenoids from Heterobranch molluscs: (**48**) amphilectene; (**49**) kalihinol A; (**50**) pustulosaisonitrile-1; (**51**) glandulaurencianol A; (**52**) punctatol; (**53**) dictyol B; (**54**) aplysin-20; (**55**) aplysiadiol; (**56**) aplykurodin A; (**57**) dolabellol A.

Nudibranchs, pleurobranchoids, sea hares, and pulmonates possess bicyclic diterpenoids. In sea slugs, *Phyllidiella pustulosa* from China represents the first finding of isocyanide diterpenoids in the family, some of them previously identified from *Acanthella* sponges, thus confirming the prey–predator relationship [126,127,128]. These diterpene isonitriles are kalihinene, amphilectene (48), kalihinol-A (49), and kalihinol-E (Figure 7). *P. pustulosa* from Vietnam also contains these compounds and some sterols [129]. Three isonitriles, pustulosaisonitriles-1 (50), -2, and -3 (Figure 7) are also found in *P. pustulosa* from Australia, with pustulosaisonitrile-1 exhibiting moderate levels of antimalarial activity *in vitro* [188]. The aeolid *Spurilla* sp. from Argentina presents spurillin B together with other compounds [183].

In pleurobranchoids, *Pleurobranchaea meckelii* from the Mediterranean possesses two labdane aldehyde diterpenes in the mantle [189], which are similar to those of the pulmonate *Trimusculus reticulatus* (see below).

Regarding sea hares, *Aplysia punctata* presents three brominated diterpenes, glandulaurencianols A–C (51) (Figure 7), from the red algae *Laurencia glandulifera* [190], as well as punctatol (52) (Figure 7) [191]. All these compounds possess a laurencianol skeleton, a known antibacterial diterpene from the red algae *L. obtusa* [192]. Similarly, the Atlantic and Mediterranean *A. depilans* present the guanidine diterpenes dictyol-A and B (53) (Figure 7), from the brown algae *Dictyota coriacea* which they feed upon [193], while the Atlantic sea hares also present steroids and peroxy sterols [194,195]. *A. kurodai* presents many halogenated and brominated mono- and diterpenes distributed in different body parts. These include aplysin-20 (54), isoaplysin-20, aplysiadiol (55), epi-aplysin-20, and *ent*-isoconcinndiol (Figure 7) [196,197,198], derived from isoconcinndiol of the red algae *L. snyderae* [199], as well as some monoterpenoids (see above). *A. kurodai* may also present aplykurodin A (56) and B [200] (Figure 7), as well as several alkaloids and other compounds [3]. Dolabellanes [81,82] from brown Dictyotaceae algae are found in *Dolabella auricularia* from the Gulf of California, while in the Indian Ocean it presents the similar dolatriol, together with the monoterpenoid (−)−loliolide [83,84]. *Dolabella auricularia* is also the source of the bromo-chloro-diterpenoid, dolabellol A (57) (Figure 7) [201].

The pulmonate genus *Trimusculus* does not possess the typical propionates common in this group, instead it possesses a single type of labdane diterpenoids, some of them similar to those of the pleurobranchoid *Pleurobranchaea meckelii* [189]. The South African *T. costatus* possesses the labdanes 6β,7a-diacetoxylab-8,13-dien-15-ol and 2α,6β,7a-triacetoxylabda-8,13-dien-15-ol, which are toxic to *Artemia salina* and inhibit feeding of the predatory fish *Pomadasys commersonnii* [202]. The New Zealand *T. reticulatus* secrets several diterpenes from its mantle and foot that are deterrents against sea stars [203]. Similarly, related species from Chile and South Africa, such as *T. costatus* and *T. peruvianus*, present cytotoxic and anti-feedant activities [202,204,205,206].

### 4.4. Tricyclic Diterpenoids

**Figure 8:**
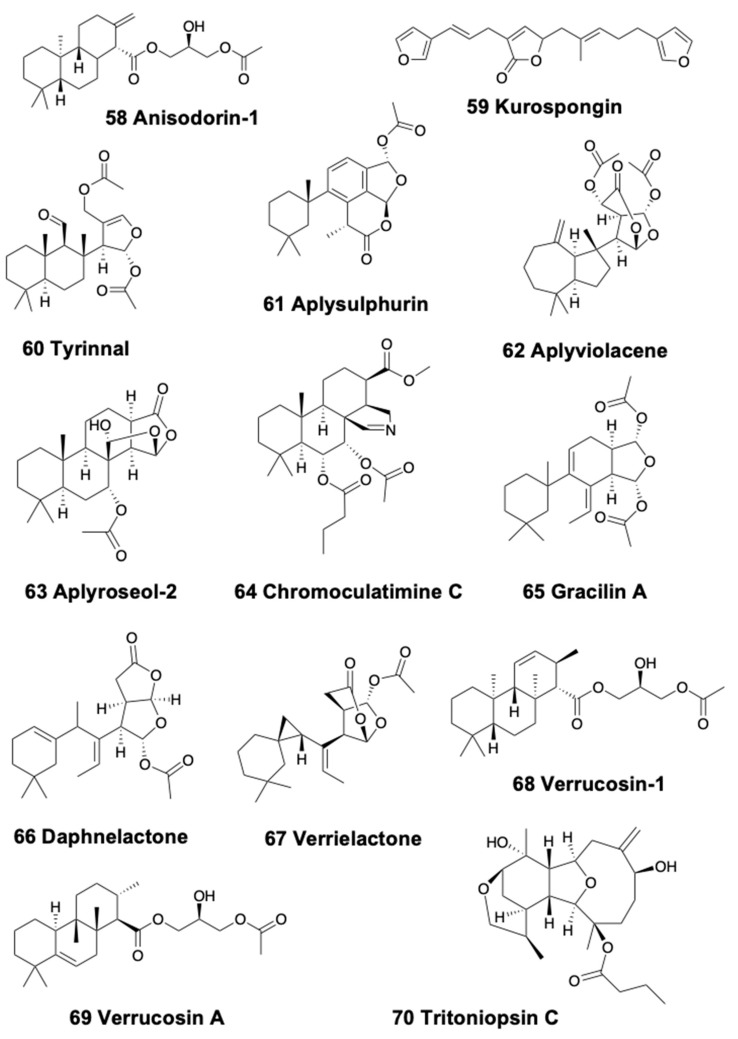
Structures of selected diterpenoids from Heterobranch molluscs: (**58**) anisodorin-1; (**59**) kurospongin; (**60**) tyrinnal; (**61**) aplysulphurin; (**62**) aplyviolacene; (**63**) aplyroseol-2; (**64**) chromoculatimine C; (**65**) gracillin A; (**66**) daphnelactone; (**67**) verrielactone; (**68**) verrucosin-1; (**69**) verrucosin A; (**70**) tritoniopsin C.

Tricyclic diterpenoids are present in sea slugs, pleurobranchoids, and sea hares. The Patagonian doridacean *Anisodoris fontaini* contains anisodorins 1–5 (58) (Figure 8), which are said to be *de novo* biosynthetized [207]. Within nudibranchs, *Chromodoris* species (now called *Felimida* in Eastern Pacific, Atlantic, and Mediterranean species, and different genera for other geographical areas) [149], usually present a wide variety of diterpenes obtained from their sponge preys accumulated into MDFs situated in their mantle border [15]. The South African chromodorid *Chromodoris hamiltoni* presents four unusual chlorinated homoditerpenes, the hamiltonins A–D, among other compounds [208]. *Chromodoris africana* from the Red Sea, instead, contains the furanoterpene kurospongin (59) (Figure 8) and a macrolide. Kurospongin was also found in an Okinawan sponge, *Spongia* sp., displaying ichthyotoxicity and feeding deterrency [209,210,211]. Tyrinnal (60), an unusual diterpene seco-11,12-spongiane (Figure 8), is found together with some known sesquiterpenes in the Patagonian chromodorid *Tyrinna nobilis* [89]. *Goniobranchus albopunctatus* (previously known as *Chromodoris albopunctata*) from Australia presents 12α-acetoxyspongian-16-one, 20-acetoxyspongian-16-one, (+)-spongian-16-one, and 7α-acetoxyspongian-16-one, together with some propionates and (+)-isoagatholactone [90]. The same study reports that *Chromodoris splendida* from Australia presents aplysulphurin (61) (Figure 8), probably from its diet, the demosponge *Darwinella tango*, together with gracilin metabolites, such as gracilin C 20, not found in the sponge. A recent study on 19 species of Chromodoridid nudibranchs from Australia, mentioned above, reported many diterpenoids, among other compounds, found in mantle and internal glands [154]. In that work, mixtures of compounds are described in *Goniobranchus* nudibranchs, containing spongian diterpenes, norditerpenes, and rearranged diterpenes. *G. tinctorius* contains two rearranged diterpenes, aplysulphurin and aplyviolacene (62) (also named Macfarlandin E; Figure 8) in the viscera and four spongian diterpenes in the mantle. *G. tasmaniensis* contains the spongian diterpene aplyroseol-2 (63) (Figure 8), together with two related dialdehydes in the mantle, and other terpenes in the viscera, of the aplyroseol/dendrillol type. *G. collingwoodi* possesses a series of 12 diterpenes, including 10 spongian diterpenes, and the furanone diterpene luffarin-X [154,212]. Moreover, *G. splendidus* presents 31 different terpenes in mantle and viscera, 22 of which are regular spongian diterpenes, including chromoculatimine C (64) (Figure 8), 6α,7α-diacetoxydendrillol-3, and methyl 15,17-epoxy-7,17α-diacetoxy-ent-isocopalan-16-oate, while six compounds, aplysulphurin, membranolide, aplyviolene, tetrahydroaplysulphurin-1, tyrinnal, and splendidalactone-1 present a rearranged diterpene skeleton, and two compounds present a rearranged norditerpene (C19) skeleton of the gracilane type [154,213]. Thus, *G. splendidus* presents spongian diterpenes, rearranged diterpenes and norditerpenes, especially in the mantle rim, but populations differ in abundance, type, and richness of these compounds, also displaying a high individual variation between specimens from the same population, perhaps related to variability in dietary sponge communities among geographic locations [214]. *G. daphne* presents the norditepenes gracilin A (65) and daphnelactone (66) (Figure 8) in mantle and viscera, while the rearranged diterpene aplysulphurin is only present in the viscera [154]. *G. hunterae* also contains aplysulphurin, as well as aplyviolene, and aplyviolacene (Macfarlandin E), while *G. verrieri* presents the spongian norditerpene verrielactone (67) (Figure 8) and the rearranged diterpenes dendrillolide A and aplyviolacene, as well as gracilin A [154,213]. Finally, this study reports that *Ardeadoris egretta* also contains regular diterpenes with a spongian framework, with five compounds in the viscera and six in the mantle, while *Doriprismatica atromarginata* presents also spongian furanoditerpenes, three distributed in viscera and four in the mantle.

A recent chemical study of the South China Sea nudibranchs *Phyllidiella pustulosa* and *Phyllidia coelestis*, as well as their possible sponge prey *Acanthella cavernosa*, led to the isolation of several terpenoids, including a kalihinane-type diterpenoid, bisformamidokalihinol A, along with other three diterpenoids [130]. A series of amphilectene diterpenes, most likely of dietary origin, with isocyano and formamido functionalities has been recently reported in *Phyllidia coelestis* from South China [215]. The dorid *Hexabranchus sanguineus* from South China also presents a diterpene in its internal tissues, as well as other compounds probably from its diet sponge [142].

Some natural products of heterobranchs, particularly in sea slugs and sea hares, include different combinations of terpenoids with glycerides, guanidines, and others. These include the diterpene glycerides found in several doridacean slugs, such as the Mediterranean *Doris verrucosa*, possessing the verrucosins 1–9 (68) (Figure 8) [15,216] and a further series of diterpenoid glycerides. Among these compounds, verrucosin A (69) (Figure 8) is de novo biosynthesized, as demonstrated in experiments with ^13^C- and ^14^C-labelled precursors [217,218,219]. Biosynthesis is commonly found in this group [3]. Another example is the doridacean *Archidoris pseudoargus*, which possesses several ichtyotoxic diterpene glycerides in mantle and egg masses in UK specimens [220]. Similarly, *Doris* (*Austrodoris*) *kerguelenensis* from several Antarctic locations, presents a series of diterpene diacylglycerides in the mantle, which effectively protect them from predation by sympatric sea stars and anemones, along with the corresponding monoacylglycerides, and monoacylglycerides of regular fatty acids [15,106,221,222]. Additional diterpene glycerides and clerodane diterpenes, such as palmadorins, were described in specimens of *D. kerguelenensis* from diverse Antarctic populations [223,224]. The existence of different chemotypes in *D. kerguelenensis* indicates that different terpene synthases may be regulating the biosynthesis of this wide arsenal of terpene glycerides [225], and this may be related to their genetic variability and/or cryptic speciation [224,225,226,227]. Palmadorin A, B, D, M, N, and O inhibit human erythroleukemia cells (HEL) at low micromolar IC_50_, while palmadorin M inhibits Jak2, STAT5, and Erk1/2 activation in HEL cells and produces apoptosis at 5mM [224].

The aeolids *Phyllodesmium briarieum*, *P. longicirrum* and *P. magnum* possess several diterpenes along with some sesquiterpenes, possibly obtained from their octocoral prey [182,228]. Further studies report four polycyclic chatancin diterpenes in Australian specimens of *P. longicirrum,* along with other deterrent compounds against fish [229,230]. Tritoniopsins A–D (70) (Figure 8) are found in the dendronotid *Tritoniopsis elegans* in China, from its coral prey *Cladiella krempfi* [86,231]. The arminid *Dermatobranchus ornatus* from China possesses a calicophirin diterpenoid in the mantle, suggested to be obtained from its potential gorgonian prey *Muricella* sp. in South China [232]. *D. ornatus* also presents four eunicellin diterpenes, where two of them are suggested to be obtained from its prey, the gorgonian *Muricella sinensis* [46], and another one was isolated earlier from an unidentified Pacific soft coral [233]. These compounds display moderate cytotoxicity and inhibition of cell division in fertilized starfish eggs, but their ecological role is not described [46]. Similar briarane diterpenoids are known in the Mediterranean *Armina maculata* and its prey, the pennatulacean octocoral *Veretillum cynomorium* [234,235,236].

The pleurobranchoids *Pleurobranchus albiguttatus* and *P. forskalii* from Philippines possess several cytotoxic chlorinated diterpenes (chlorolissoclimide, dichlorolissoclimide, haterumaimide D, H, L, M, 3ß-hydroxylissoclimide), derived from the bioaccumulation and biotransformation of compounds from its ascidian prey, *Lissoclinum* [237].

Finally, the Chinese sea hare *Aplysia argus* presents derived *Laurencia* algal compounds, i.e. cyclopropane and cyclobutane rings, in five brominated pimarane diterpenoids, biotransformed by adding acetoxy groups [238].

### 4.5. Tetracyclic Diterpenoids

**Figure 9:**
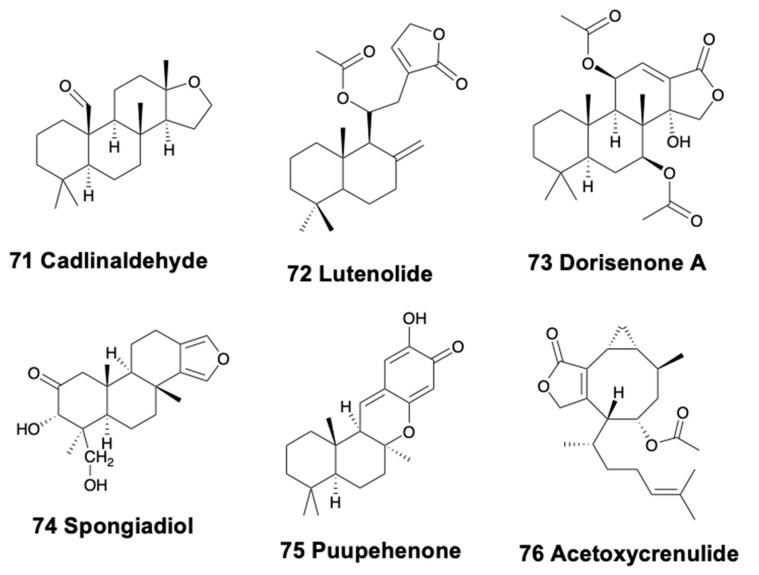
Structures of selected diterpenoids from Heterobranch molluscs: (**71**) cadlinaldehyde; (**72**) lutenolide; (**73**) dorisenone A; (**74**) spongiadiol; (**75**) puupehenone; (**76**) acetoxycrenulide.

Only doridacean slugs and one sea hare contain tetracyclic diterpenoids. In British Columbia, *Cadlina luteomarginata* presents five diterpenoids, cadlinaldehyde (71), spongian, secospongian, 20-acetoxy-12-marginatone, and lutenolide (72) (Figure 9) in their external extracts, some of them resulting from degradation of other terpenoids. This species is reported to be able to biosynthesize some of its terpenoids [147,148]. *Chromodoris hamiltoni* from Mozambique presents two spongiane diterpene lactones, 7β,11β-diacetoxy-16-oxospongian-17-al and 7β,11β-diacetoxy-16-oxospongi-12-enal, along with the macrolide latrunculin B [208,239]. The Japanese slug *Chromodoris obsoleta* possesses seven cytotoxic sponge diterpenoids, the dorisenones A–D (73) (Figure 9), and some related compounds [240]. The slug *Chromodoris petechialis* from Hawai’i presents three sponge diterpenes, probably from its diet sponge *Chelonaplysilla* sp. [152], one of them being cytotoxic [240]. *Chromodoris mandapamensis* from India contains spongiadiol (74) (Figure 9), which is also present in *Glossodoris cincta* (cited as *G. atromarginata*) from Egypt, and was previously isolated from Australian sponges, within a mixture of related spongiane compounds [170]. *C. mandapamensis* was found on an unidentified sponge also containing spongiadiol, and thus supporting its dietary origin. Spongiadiol displayed antiviral activity against herpes simplex virus [241]. *Chromodoris elisabethina* from Australia contains the oxygenated diterpene puupehenone (75) (Figure 9), known as cytotoxic and with antitubercular activity but with unknown ecological activity [90]. *Glossodoris cincta* from Egypt presents five spongian diterpenes in its digestive gland and dorsal mantle [170,242,243], which are also found in specimens from Sri Lanka [46,244]. Some of these metabolites have been reported to be cytotoxic and antiviral in the laboratory [241,245]. *C. kunei* from Okinawa possesses a cytotoxic spongian diterpene, probably also from its diet sponge *Dysidea* cf. *arenaria* [246]. *C. reticulata* from Australia provided six oxygenated diterpenes, two of which contain cyclic imine functionality, together with seventeen known diterpenes more, among which aplyroseol-2 was the major compound in the mantle along with some dialdehydes [175].

Regarding the only example from sea hare, in the Eastern Pacific, *Aplysia vaccaria* contains in its digestive gland several ichthyotoxic non-halogenated diterpenoids, crenulides (76) (Figure 9), derived from its diet, the brown algae *Dictyota crenulata* [247,248].

## 5. Sesterterpenoids

Sesterterpenoids possess 25 carbons, five isoprene units, and they originate from GFPP. Biogenetically, sesterterpenoids are derived from five DMAPP monomers that form the C25 GFPP backbone (10). Cyclisation of GFPP is catalysed by sesterterpene synthases. They are rare in nature relative to other types of terpenes. In Heterobranch molluscs, only linear, bicyclic, and tetracyclic sesterterpenes have been reported, and in all cases, they are found in nudibranchs (Table 4).

### 5.1. Linear Sesterterpenoids

**Figure 10:**
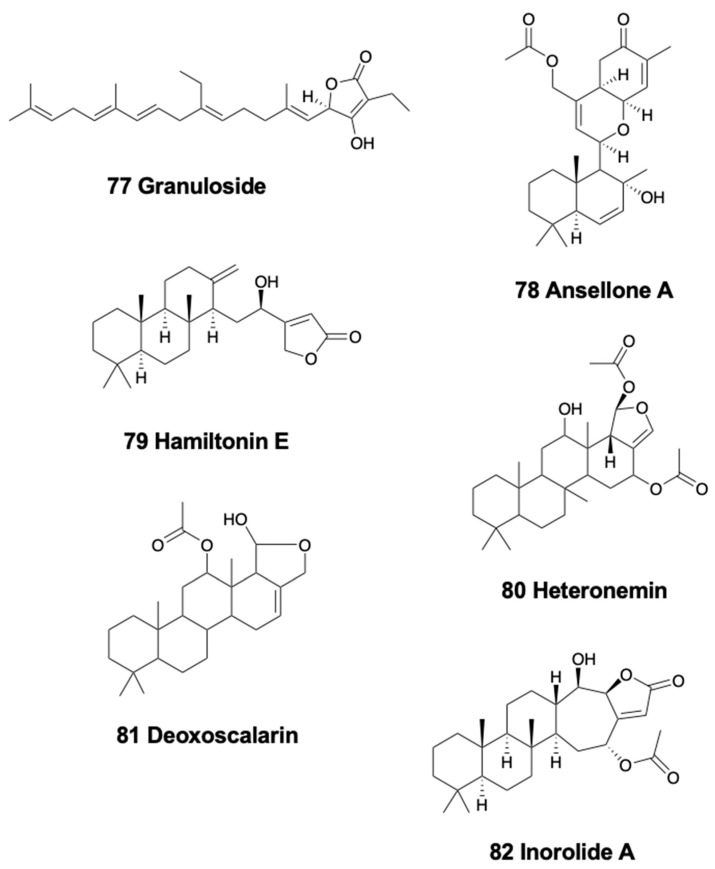
Structures of selected seterterpenoids from Heterobranch molluscs: (**77**) granuloside; (**78**) ansellone A; (**79**) hamiltonin E; (**80**) heteronemin; (**81**) deoxoscalarin; (**82**) inorolide A.

Only one nudibranch species has been described to possess linear sesterterpenoids. Interestingly enough, the Antarctic cladobranch slug *Charcotia granulosa* presents the unique linear homosesterterpene, granuloside (77) (Figure 10) [249], suggested to be *de novo* biosynthesized and stored in MDF-like structures, and probably released as a deterrent [250].

### 5.2. Bicyclic Sesterterpenoids

The only species possessing bicyclic sesterterpenoids is the South African chromodorid *Hypselodoris capensis*, which presents the 22-deoxy-23-hydroxymethyl-variabilin, together with the abovementioned compounds, obtained from its suggested prey demosponges *Fasciospongia* sp. and *Dysidea* sp. [151].

### 5.3. Tetracyclic Sesterterpenoids

This type of sesterterpenoids is only found in chromodorid nudibranchs. *Cadlina luteomarginata* from Canada and its prey, the demosponge *Phorbas* sp., both possess the sesterterpenoid ansellone A (78) (Figure 10), with unknown ecological role, but activating the cAMP signaling pathway [251]. The South African *Chromodoris hamiltoni*, a part of the compounds already mentioned above, presents the sesterterpene hamiltonin E (79) (Figure 10), together with some macrolides (latrunculins A and B) [208]. The Indian *Glossodoris atromarginata* and its prey, the demosponge *Spongia* sp. present two scalaranes in their mantle dermal formation-like structures [242]. Moreover, these sea slugs were found on two other potential prey, the sponge *Hyattella cribriformis* presenting pentacyclic scalaranes, and an unidentified sponge, possibly *Spongia* sp., possessing heteronemin (80) (Figure 10) and other scalaranes [46,170,242]. Heteronemin was further reported to display antimycobacterial activity towards *Mycobacterium tuberculosis* H_37_Rv with an MIC of 6.25 μg/ml [252]. Deoxoscalarin (81) (Figure 10), previously reported in a Mediterranean sponge, was found in *G. atromarginata* and the sponge *H. cribriformis*, supporting its dietary origin. The specimens found on *Spongia* instead, presented heteronemin, known from the sponge *Heteronema erecta*, as well as two scalaranes, one of them also reported from an unidentified sponge. Furthermore, *G. rufomarginata* from China presents scalarane compounds derived from feeding on an unidentified sponge, which are biotransformed into scalaradial derivatives. In particular, scalaradial, a potent anti-inflammatory metabolite [253], and its 12-deacetyl derivative are present in the sponge, while the absence of scalaradial in the slug supports their ability to transform this toxic compound into related scalaranes [254]. Similarly, *G. pallida* specimens from China, as well as *G. vespa* and *G. averni* from Australia, contain 12-deacetoxy-12-oxoscalaradial [255], while *G. pallida* from Guam presents different sesquiterpenes including scalaradial, deacetylscalaradial, and deoxoscalarin; these compounds are used as feeding deterrents against sympatric crabs and reef fish and are located in the mantle border of the slugs [256,257]. Scalaradial and deacetylscalaradial are also present in the demosponge *Cacospongia* sp., which is preyed upon by *G. pallida*. As reported above, *Glossodoris* species probably biotransforms their dietary scalaranes into related molecules in a detoxification process. Supporting this, the injection of scalaradial in the viscera of *G. pallida* was reported to be not toxic for the slug but resulted instead in rapid transformation of the metabolite in less than 24 h [3]. All these data support the dietary acquisition and further biotransformation of the scalarane compounds in chromodorid nudibranchs [46,170]. On the other hand, *Glossodoris sedna* from Costa Rica possess several scalarane sesterterpenes, 12-deacetyl-23-acetoxy-20-methyl-12-epi-scalaradial, 12-deacetyl-23-acetoxy-20-methyl-12-epi-deoxoscalarin, and 12-deacetyl-20-methyl-12-epi-deoxoscalarin, the first of which moderately inhibits mammalian phospholipase A2 (IC_50_ 18 μM) and is ichthyotoxic at 0.1 ppm against the allopatric fish *Gambusia affinis* [258]. Contrastingly, specimens of *G. dalli* from the same place contain the scalarane sesterterpenes 20-deoxoscalarin and 12-epi-20-deoxoscalarin [258]. Additionally, in Japan, *Chromodoris inornata* contains three cytotoxic sesterterpenes, inorolides A (82), B, and C (Figure 10), and five scalaranes [259]. Recently, *Glossodoris hikuerensis* and *G. vespa* from Australia have been reported to contain heteronemin in the viscera, while scalaradial, 12-deacetoxy-12-oxoscalaradial, and 12-deacetoxy-12-oxo-deoxoscalarin are present in the mantle [154].

## 6. Triterpenoids

Triterpenoids consist of six isoprene units (three terpene units) with the molecular formula C_30_H_48_. The linear triterpene squalene is derived from the reductive coupling of two molecules of FPP and is the precursor to all steroids [6], as mentioned above. Triterpenes exist in a great variety of structures. Nearly 200 different skeletons have been identified in nature [6]. These skeletons may be broadly divided according to the number of rings present. In general, pentacyclic structures tend to dominate in the organisms. Triterpenes are biosynthesized through the head-to-head condensation of two FPP units to form a squalene. In turn, the squalene serves as a precursor for the formation of triterpenoids, including eukaryotic sterols. The squalene synthase is a prenyltransferase that catalyzes a complex series of cationic rearrangements to join the C-1 carbons of two farnesyl residues. Differently from other marine invertebrates, such as echinoderms, heterobranch molluscs do not seem to present saponins (triterpenoid glycosides). As far as we know, no linear triterpenoids are described in heterobranchs, while cyclic triterpenoids are present only in nudibranchs, pleurobranchoids, and anaspideans (Table 5).

**Figure 11:**
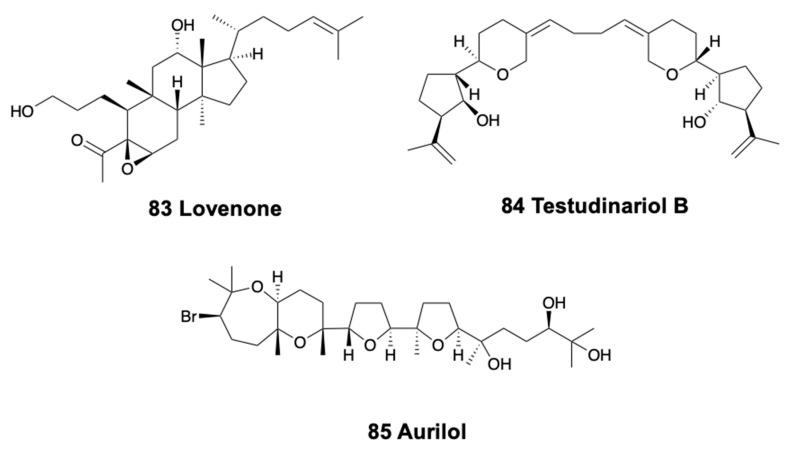
Structures of selected triterpenoids from Heterobranch molluscs: (**83**) lovenone; (**84**) testudinariol B; (**85**) aurilol.

The doridacean nudibranch from the North Sea, *Adalaria loveni*, contains lovenone (83) (Figure 11), a cytotoxic degraded triterpenoid suggested to come from an unidentified bryozoan prey [260]. Testudinariols A and B (84) (Figure 11), from the Mediterranean pleurobranchoid *Pleurobranchus testudinarius* are present in the mantle and mucus and are similar to some sponge compounds [261] although they are thought to be biosynthesized. Testudinariols are chemically related to limatulone of the limpet *Lottia limatula* [262]. Similarly, an unidentified species of *Pleurobranchus* from the South China Sea also possesses testudinariol B [263].

In sea hares, aplysiols A and B are tetracyclic triterpene polyethers found in the mantle of *Aplysia argus* from China, similar to compounds from the red algae *Laurencia* [264]. On the other hand, *Dolabella auricularia* modifies its molecules from dietary brown and red algae, while *de novo* synthesizing polypropionates and peptides [265]. Among the metabolites derived from red algae, the polyether bromotriterpene aurilol (85) (Figure 11) is cytotoxic to the HeLa tumor cell line [266].

## 7. Carotenoids (Tetraterpenoids)

Carotenoids are tetraterpenoids produced by plants and algae, as well as several bacteria and fungi. All carotenoids are derivatives of tetraterpenes, and are thus produced from 8 isoprene molecules (four terpene units); they contain 40 carbon atoms. Tetraterpenes are produced by the head-to-head union of two molecules of GGPP, similar to the formation of a squalene [267]. In general, carotenoids absorb wavelengths from 400 to 550 nm (violet to green light). This causes these compounds to be characteristically colored in yellow, orange, or red. The general structure of the carotenoid is a polyene hydrocarbon chain consisting of 9–11 double bonds and sometimes terminated by rings, with or without additional oxygen atoms attached. This structure of conjugated double bonds leads to a high reducing potential [268]. When carotenoids are present in heterobranchs, they are always diet derived (Table 6). Although many heterobranchs probably possess carotenoids, very few studies have properly described them. Eight carotenoids and 22 polyunsaturated fatty acids, all of them with anti-inflammatory potential, have been found in the digestive gland of the sea hare *Aplysia depilans* [269]. These carotenoids are obtained from the algae they feed upon.

## 8. Steroids

Steroids consist of four rings arranged in a specific molecular configuration. They are important components of cell membranes altering membrane fluidity, and may also act as signaling molecules. Hundreds of steroids are found in plants, animals, and fungi. In animals, all steroids are manufactured in cells from lanosterol, which is derived by cyclization of the triterpene squalene [9]. They are typically composed of seventeen carbon atoms, bonded in four fused rings: three six-member cyclohexane rings and one five-member cyclopentane ring. Steroids vary by the functional groups attached to this four-ring core and by the oxidation state of the rings. Sterols are steroids with a hydroxy group at position 3 and a skeleton derived from cholestane [9]. 

**Figure 12:**
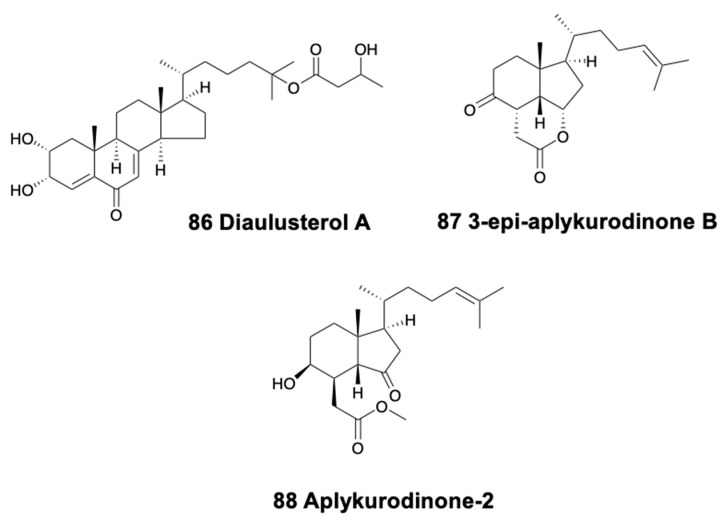
Structures of selected steroids from Heterobranch molluscs: (**86**) diaulusterol A; (**87**) 3-epi-aplykurodinone B; (**88**) aplykurodinone-2.

Only nudibranchs and anaspideans are reported to present steroids (Table 6); many other heterobranchs probably do so as well, albeit they have not been investigated. Among sea slugs, the doridacean *Aldisa smaragdina* from Spain presents a progesterone homologue in their external tissues [270]. The related species *Aldisa sanguinea* was previously reported to have similar steroids and to use them against predators [15]. The dorid *Doris* aff. *verrucosa* from Brazil presents a variety of common sterols in the mantle [271], and another *Doris (Archidoris)* sp. also possess similar glycerids, some of them *de novo* biosynthesized [15,272]. On the other hand, among the caryophyllidid doridoidea, diaulusterol A (86) (Figure 12) is partially biosynthesized by *Diaulula sandieguensis* [273]. *Phyllidiella pustulosa* from Vietnam also contained some sterols [129]. Moreover, the aeolidacean species *Cratena peregrina*, *Flabellina affinis*, and *Flabellina (Coryphella) lineata* present several hydroxy and acetoxysterols from their *Eudendrium* hydroids diet in the Mediterranean [274].

In sea hares, *Aplysia fasciata* from the Mediterranean possesses certain ichthyotoxic degraded sterols, like 4-acetylaplykurodin-B, aplykurodinone B, and 3-*epi*-aplykurodinone B (87) (Figure 12) [275], in their external tissues, similar to the steroids found in the Atlantic specimens of the same species [276]. These steroids are closely related to aplykurodin B found in the Pacific species *A. kurodai* [200]. In addition, two secosterols have been found in the sea hare *A. kurodai* [277]. Mucus secretions in both Atlantic and Mediterranean specimens of *A. punctata* present epidioxy sterols, as those of *A. depilans* found in its digestive gland [195]. 

*Syphonota geographica,* a Mediterranean-invasive sea hare, presents two degraded sterols, aplykurodinone-1 and -2 (88) (Figure 12), in the mantle [278]. In their digestive gland, though, they present a dietary macrocyclic glycoterpenoid, syphonoside, derived from the sea grass *Halophila stipulacea* [279]. Syphonoside might be the precursor of several terpenoids found exclusively in *S. geographica*, thus suggesting its biotransformation from a seagrass diet.

## 9. Concluding Remarks

Heterobranch molluscs are rich in natural products; however, only a small proportion has been studied so far. As reported here, their terpenoids are particularly abundant and diverse; they include monoterpenoids, sesquiterpenoids, diterpenoids, sesterterpenoids, triterpenoids, tetraterpenoids, and steroids. This review discusses more than 330 metabolites isolated from ca. 70 species of heterobranch molluscs. The monoterpenoids reported here may be linear or monocyclic, while sesquiterpenoids may include linear, monocyclic, bicyclic, or tricyclic molecules. Diterpenoids may include linear, monocyclic, bicyclic, tricyclic, or tetracyclic compounds. Sesterterpenoids, instead, are linear, bicyclic, or tetracyclic. Triterpenoids, tetraterpenoids, and steroids are not as abundant as the previously mentioned types. Remarkably, no terpenoids have been described in this period in tylodinoideans, cephalaspideans, or pteropods; and most terpenoids have been found in nudibranchs, anaspideans, and sacoglossans, with very few compounds in pleurobranchoideans and pulmonates. Many of these compounds found in heterobranchs are obtained from their diet, while others are biotransformed, or *de novo* biosynthesized by the molluscs. Bacterial origin has been suggested for several types of compounds in heterobranchs [280]. However, very few studies rigorously demonstrate this. The structural similarity and dietary origin of some natural products from microorganisms (particularly cyanobacteria) indicate that this is possible, but further studies are required to shed light on this very interesting topic.

Monoterpenoids are present mostly in anaspidea and are less abundant in sacoglossa. Nudibranchs are especially rich in sesquiterpenes, which are also present in anaspidea, and in less numbers in sacoglossa and pulmonata. Diterpenoids are also abundant in nudibranchs, present also in anaspidea, and scarce in pleurobranchoidea, sacoglossa, and pulmonata. Sesterterpenoids are only found in nudibranchia, while triterpenoids, carotenoids, and steroids are only reported for a few nudibranchia, pleurobranchoidea, and anaspidea. Anaspidea include many halogenated and non-halogenated terpenoids, mostly from diet. Nudibranchs possess many sesquiterpenoids and diterpenoids, mostly from diet, but also *de novo* biosynthesized. Some compounds are present as glyceryl esters, such as verrucosins. *De novo* biosynthesis has been demonstrated in a few cases, as well as the putative symbiotic origin of some metabolites [3,15], probably related to the intrinsic difficulties of this kind of research. Dietary origin is much better established, with compounds originating from many diverse organisms, from different kinds of algae and seagrasses to metazoans, such as Porifera, Cnidaria, Mollusca, Bryozoa, Tunicata, and others. In some cases, however, it remains a hypothesis that should be further demonstrated.

The ecological role of these compounds includes many examples of feeding deterrence against sympatric predators, mostly fish and crabs, as well as toxicity. However, many assays are still conducted with species that do not live in the same habitat as the heterobranchs, and thus the ecological significance of many compounds continues to be further examined. The low numbers of reliable field experimental data available is evident. The pharmacological potential of heterobranch terpenoids is clear from the examples reported above—from cytotoxicity to antiplasmodial activity, antituberculosis, antifouling, antifungal, antibacterial, antitumoral, and apoptosis inducers.

Overall, a huge variety of terpenoid structures is found in heterobranchs, indicating that chemodiversity correlates to the biodiversity of this amazing group of molluscs. The potential to vastly increase the number and diversity of known natural products in heterobranchs, as well as their bioactivity, is open to further research. The search for effective and efficient methods of integral and/or selective extraction of terpenoids from heterobranchs, in particular, deserves more attention, since environmentally friendly practices are needed. How global change will affect biodiversity, and thus chemodiversity, in heterobranch molluscs remains to be further investigated, hopefully before both species and molecules disappear.

## Figures and Tables

**Table 1:** Number of monoterpenoids in the different Heterobranch groups in the reviewed period. In brackets: number of species with monoterpenoids.

Group (Species)/Compounds	Anaspidea (5)	Sacoglossa (11)
Linear Monoterpenoids	>10 ^1^	5
Monocyclic Monoterpenoids	>10 ^1^	2

^1^ Undetermined number of derivatives or unnamed compounds.

**Table 2:** Number of sesquiterpenoids in different Heterobranch groups in the reviewed period. In brackets: number of species with sesquiterpenoids.

Group (Species)/Compounds	Nudibranchia (51)	Anaspidea (7)	Sacoglossa (2)	Pulmonata (1)
Linear Sesquiterpenoids	1	0	0	0
Monocyclic Sesquiterpenoids	5	>5 ^1^	3	2
Bicyclic Sesquiterpenoids	>60 ^1^	>6 ^1^	0	0
Tricyclic Sesquiterpenoids	>5 ^1^	3	0	0

^1^ Undetermined number of derivatives or unnamed compounds.

**Table 3:** Number of diterpenoids in different Heterobranch groups in the reviewed period. In brackets: number of species with diterpenoids.

Group (Species)/Compounds	Nudibranchia (39)	Pleurobranchoidea (3)	Anaspidea (8)	Sacoglossa (5)	Pulmonata (3)
Linear Diterpenoids	1	0	0	11	0
Monocyclic Diterpenoids	>8 ^1^	0	10	2	0
Bicyclic Diterpenoids	8	2	>15 ^1^	0	>2 ^1^
Tricyclic Diterpenoids	>85 ^1^	7	5	0	0
Tetracyclic Diterpenoids	25	0	4	0	0

^1^ Undetermined number of derivatives or unnamed compounds.

**Table 4:** Number of sesterterpenoids in the different Heterobranch groups in the reviewed period. In brackets: number of species with sesterterpenoids.

Group (Species)/Compounds	Nudibranchia (13)
Linear Sesterterpenoids	1
Bicyclic Sesterterpenoids	1
Tetracyclic Sesterterpenoids	>16 ^1^

^1^ Undetermined number of derivatives or unnamed compounds.

**Table 5:** Number of triterpenoids in different Heterobranch groups in the reviewed period. In brackets: number of species with triterpenoids.

Group (Species)/Compounds	Nudibranchia (1)	Pleurobranchoidea (2)	Anaspidea (2)
Cyclic Triterpenoids	1	2	3

**Table 6:** Number of carotenoids and steroids in different Heterobranch groups in the reviewed period. In brackets: number of species with carotenoids and/or steroids.

Group (Species)/Compounds	Nudibranchia (8)	Anaspidea (5)
Carotenoids	0	8
Steroids	>10 ^1^	>8

^1^ Undetermined number of derivatives or unnamed compounds.
